# Neuroprotective and Anti-Aging Potentials of Essential Oils from Aromatic and Medicinal Plants

**DOI:** 10.3389/fnagi.2017.00168

**Published:** 2017-05-30

**Authors:** Muhammad Ayaz, Abdul Sadiq, Muhammad Junaid, Farhat Ullah, Fazal Subhan, Jawad Ahmed

**Affiliations:** ^1^Department of Pharmacy, University of MalakandChakdara, Pakistan; ^2^Department of Pharmacy, University of PeshawarPeshawar, Pakistan; ^3^Institute of Basic Medical Sciences (IBMS), Khyber Medical University (KMU)Peshawar, Pakistan

**Keywords:** essential oils, Alzheimer’s disease, cholinesterase inhibitors, antioxidants, amyloid-β, NFTs, dementia, BACE1

## Abstract

The use of essential oils (EOs) and their components is known since long in traditional medicine and aromatherapy for the management of various diseases, and is further increased in the recent times. The neuroprotective and anti-aging potentials of EOs and their possible mechanism of actions were evaluated by numerous researchers around the globe. Several clinically important EOs and their components from *Nigella sativa*, *Acorus gramineus, Lavandula angustifolia, Eucalyptus globulus, Mentha piperita, Rosmarinus officinalis, Jasminum sambac, Piper nigrum* and so many other plants are reported for neuroprotective effects. This review article was aimed to summarize the current finding on EOs tested against neurodegenerative disorders like Alzheimer disease (AD) and dementia. The effects of EOs on pathological targets of AD and dementia including amyloid deposition (Aβ), neurofibrillary tangles (NFTs), cholinergic hypofunction, oxidative stress and glutamatergic abnormalities were focused. Furthermore, effects of EOs on other neurological disorders including anxiety, depression, cognitive hypofunction epilepsy and convulsions were also evaluated in detail. In conclusion, EOs were effective on several pathological targets and have improved cognitive performance in animal models and human subjects. Thus, EOs can be developed as multi-potent agents against neurological disorders with better efficacy, safety and cost effectiveness.

## Introduction

Essential oils (EOs) represent a mixture of highly complex, naturally occurring, volatile compounds synthesized by plants as secondary metabolites. The EOs are abundant in flowers, leaves, seeds, rhizomes, barks and are usually isolated via hydro-distillation, cold pressing methods (Edris, 2007). A most frequently used laboratory method is steam distillation which was developed in the Middle

Ages by Arabs. The use of EOs as therapeutic remedy is very ancient and in the bible, EOs were considered as spiritual, mental and physical healing agents (Guenther, 1950). Hippocrates, a prehistoric Greek physician, named the aromatic plants as “father of medicines” and these plants containing EOs were used as food preservatives, flavoring agents and for medicinal purposes. The term “essential oil” was described by component of a drug as “Quinta essential” in his book (Gaysinsky and Weiss, 2007). These days, EOs are used in traditional medicines, alternative medicines, Chinese medicines, aromatherapy, massage therapies, as well as in cosmetics, perfumes and food industries (Smith-Palmer et al., 2001; Burt, 2004). In Egypt, EOs extracted from aromatic plants were employed for the prevention and treatment of various diseases. Soon after, the Greeks and Romans adopted Egyptian practices of using EOs in aromatherapy and to improve their quality of life. For instance, they employed steam baths infused with oils of jasmine, ylang-ylang and lavender for central nervous system (CNS) stimulation and mental relaxation (Ballard et al., 2002; Keville and Green, 2012).

Naturally, EOs play a vital role in plants protection and propagation by acting as antimicrobials, repellent for herbivores and attractive agents for insects which aid in pollination. Owing to their natural properties, EOs have been largely used as antibacterial, antifungal and insecticidal agents. Greater understandings of EOs chemistry and penetrative capabilities via biological membranes have led them as important treatment tools for the management of various neurological disorders. Presently, about 3000 EOs are known, among which 300 are commercially vital particularly for food, pharmaceutical, cosmetic, agronomic, sanitary and perfume industries (Arctander, 1969; Pichersky et al., 2006). For instance, _D_-carvone, _D_-limonene and geranyl acetate are used in the preparations of creams, perfumes, soaps, as flavoring agents in food products, as fragrances for domestic cleaning products and as industrial solvents (Hajhashemi et al., 2003; Silva et al., 2003). Furthermore, EOs in combination with vegetable oils are used in massages (Cooke and Ernst, 2000). Some EOs appear to reveal medicinal properties and are reported to cure one or more diseases and are used in Para-medicinal practices (Perry et al., 2003).

## Chemistry

**Figure 1:**
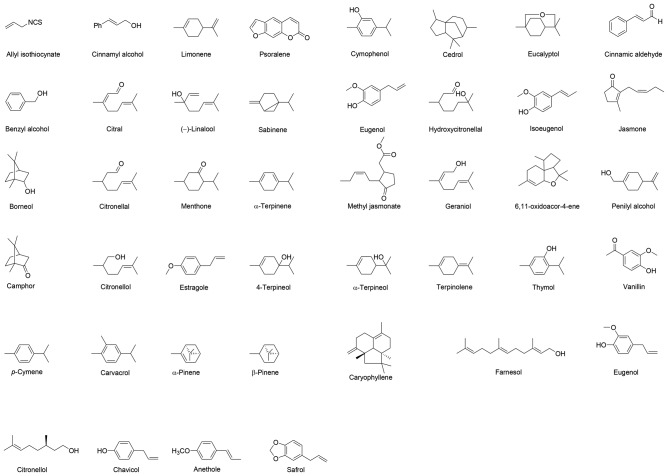
Chemical structures of most abundant and therapeutically active compounds in essential oils (EOs).

EOs comprise of extremely complex mixtures of compounds, and contain numerous components in variable concentrations (Figure 1). The fundamental components of EOs are aromatic compounds, terpenes/terpenoids and aliphatic molecules, particularly with low molecular weights (Burt, 2007; Bakkali et al., 2008; González-Burgos et al., 2011). Specific EOs have usually higher concentrations (20%–95%) of two or three components, whereas, other components are present in minor amounts. For instance, linalol represent 68% of the *Coriandrum sativum* EO content, whereas, alpha/beta thuyone and camphor constitute 57% and 24% of *Artemisia Herba-alba* EO. Similarly, constituents with comparatively higher concentrations are _D_-limonene (>80%) in citrus peel oils, carvacrol and thymol (30 and 27%) in *Origanum compactum*. Similarly, α-phellandrene and limonene (36 and 31%) in *Anethum graveolens* leaf EO, 1,8-cineole (50%) in *Cinnamomum camphora* EO, menthol and menthone (59 and 19%) in *Mentha piperita* EO are the major components. The chemical composition profile of EOs vary in the number of molecules and stereochemical form based on the extraction techniques used, climate, plant origin, soil composition, vegetative cycle stage and age (Newall et al., 1996; Angioni et al., 2006). Therefore, to achieve EOs with uniform chemical composition, they must be extracted from the same plant’s part and harvested under the same growing conditions (Soil, water, food, climate) and most effective season. Majority of the commercialized EOs are chemically characterized via gas chromatography-mass spectrometry (GC-MS) techniques. Analytical monographs regarding the quality of EOs have been published in European Pharmacopoeia, WHO, ISO and Council of Europe (Smith et al., 2005). The main components of EOs contribute and determine the biological characteristics. Due to the development of modern analytical techniques, chemical composition of EO is easily analyzed.

## Role in Alzheimer Disease and Dementia

### Cholinesterase Inhibitory Potentials

**Figure 2:**
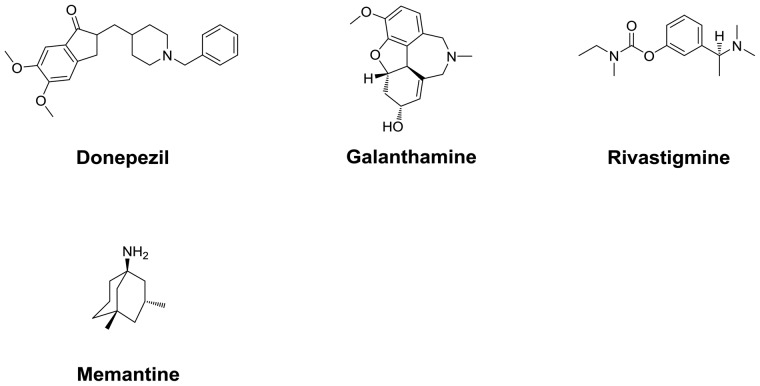
Clinically available anti-Alzheimer drugs. Donepezil, Galanthamine, Rivastigmine and Tacrine are cholinesterase inhibitors whereas, Memantine is N-methyl-d-aspartate (NMDA) receptor antagonist.

**Figure 3:**
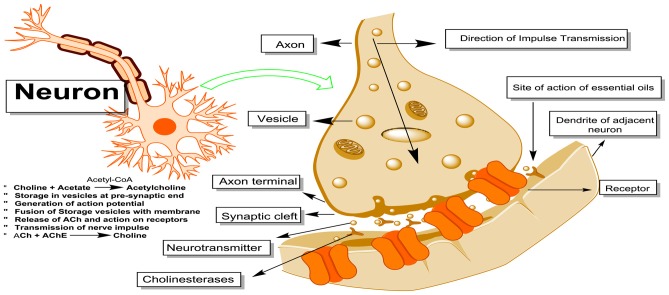
Neuronal synthesis of acetylcholine (ACh). ACh is stored in the vesicles and subsequent to action potential they get fused with the membrane and release the ACh at neuronal junction. After their action on cholinergic receptors they are enzymatically cleaved by acetylcholinesterase (AChE) and butyrylcholinesterase (BChE). EOs can inhibit the action of these cholinesterase’s and can restore their action for prolong time. Thus they are useful for the symptomatic management of Alzheimer disease (AD).

Alzheimer’s disease (AD) is the most prevalent of the neurodegenerative disorders, characterized by cognitive dysfunctions, behavioral turbulence, gradual memory loss, scarcity in cholinergic neurotransmission, oxidative stress, accumulation of amyloid plaques (amyloid-β, Aβ) and neurofibrillary tangles (NFTs) in the brain areas (Nussbaum and Ellis, 2003). Development of mechanism based inhibitors of the enzymes implicated in the AD is the most useful option in the anti-AD drug development. In this regard, inhibitors of acetylcholinesterase (AChE) and butyrylcholinesterase (BChE) enzymes involved in the degradation of essential neurotransmitter acetylcholine (ACh) represent major compounds approved for clinical use in the symptomatic management of AD. Among the currently approved anti-Alzheimer drugs, rivastigmine, tacrine, galanthamine and donepezil (4/5) are AChE inhibitors whereas, 5th one memantine, is a glutamatergic system modifier (Figure 2; O’Brien and Ballard, 2001; Reisberg et al., 2003). These cholinesterase inhibitors reversibly bind to the active sites of AChE/BChE enzymes and thus inhibit the hydrolytic degradation of an important neurotransmitter ACh, implicated in the neurotransmission (Figure 3). Consequently, the synaptic ACh concentration is augmented resulting in the relief of AD symptoms. Among the cholinesterase inhibitors, rivastigmine is a peripheral BChE inhibitor and hence associated with peripheral cholinergic side effects (Birks and Grimley Evans, 2015). Galanthamine, another natural product based cholinesterase inhibitor was originally isolated from snowdrop belonging to the Amaryllidaceae family (Heinrich and Lee Teoh, 2004).

Several studies regarding the cholinesterase inhibition potentials of EOs have been reported. Recently, Ayaz et al. (2015) reported the AChE, BChE inhibitory and free radicals scavenging efficacy of EOs from the leaves and flowers of *Polygonum hydropipe*r. They provided a comparative GC-MS analysis and identified 141 and 122 compounds in leaf and flowers oils respectively. Caryophylene oxide (41.42%) was the major component in leaf oil, whereas, decahydronaphthalene (38.29%) was major constituent of the flower oil. Leaf and flower EOs exhibited IC_50_ of 120 and 220 μg/ml respectively in AChE inhibition assays. Whereas, in BChE inhibitory assays, leaf and flower EOs revealed IC_50_ of 130 and 225 μg/ml respectively. The same EOs demonstrated significant anti-radical activities with IC_50_ of 20 and 200 μg/ml respectively in 1,1-diphenyl 2-picrylhydrazyl (DPPH) free radicals scavenging assay. The calculated IC_50_s were 180 and 60 μg/ml for leaf oil and 45 and 50 μg/ml for flower oil in 2,2-azinobis3-ethylbenzthiazoline-6-sulfonic acid (ABTS) and H_2_O_2_ anti-radicals assays respectively. The authors speculated that by targeting more than aspect of the disease with enhanced bioavailability will make these EOs more effective than currently available single-drug single-target molecules. In another study, Ahmad et al. (2016) reported the anti-cholinesterase and antiradicals potentials of EO from *Rumex hastatus* D. Don. GC-MS analysis of EO revealed the presence of 123 compounds. In Ellman assay, EO demonstrated a concentration dependent inhibition of AChE and BChE with IC_50_ values of 32.54 and 97.38 μg/ml respectively. Furthermore, in antioxidant assays using DPPH and ABTS anti-radicals protocols, the EO revealed high antioxidant efficiency with IC_50_ values of 3.71 and 6.29 μg/ml respectively (Ahmad et al., 2016). Okello et al. (2008) reported the *in vitro* AChE, BChE inhibitory activity of flower oil from *Narcissus poeticus* L. belonging to family Amaryllidaceae. GC-MS analysis revealed the presence of three main compounds including benzyl benzoate (19.0%), phenylethyl alcohol (17.5%) and benzyl alcohol (11.0%) with a series of minor components as well. At 0.1 mg/ml concentration an inhibition of 39% was observed (Okello et al., 2008). Loizzo et al. (2009) investigated the potential effectiveness of EO from *Salvia leriifolia* Benth., family Lamiaceae in the management of AD. In GC-MS analysis, camphor, cineole, camphene and alpha pinene were identified as major components with 10.5, 8.6, 6.2 and 4.7% concentration, respectively. The tested EO exhibited high BChE inhibitory, DPPH anti-radicals properties and inhibited the production of inflammatory mediators. The EO from *Marlierea racemosa* Vell. (Myrtaceae) were evaluated by Souza et al. (2009) against AChE enzyme. The chemical composition of EO was analyzed via GC-MS, indicating high concentration of spathulenol. A concentration dependent (35%–75%) and region based inhibition against AChE was observed.

Five EOs *Cistus creticus*, *Cistus monspeliensis*, *Cistus salvifolius*, *Cistus villosus* and *Cistus libanotis* from Cistaceae family, were investigated by Loizzo et al. (2013) for antioxidant and cholinesterase inhibitory potentials. EOs were characterized via GC and GC-MS analysis. Results revealed that EO from *C. monspeliensis* demonstrated most promising activity in β-carotene bleaching test with IC_50_ of 54.7 lg/mL. In FRAP assay, *C. libanotis* was found most potent with a value of 19.2 l MFe(II)/g. In cholinesterase inhibition assays, *C*. *salvifolius* exhibited AChE inhibitory activity with IC_50_ value of 58.1 μg/ml. Whereas, *C. libanotis, C. creticus* and* C. salvifolius* showed significant inhibitory activities against BChE with IC_50_ values of 23.7, 29.1 and 34.2 μg/ml respectively. Results of the study suggested that EOs from Cistus species may be developed as potential neuroprotective and AD modifying agents.

### Effect on Learning and Memory (Cognition)

Cognition is the combination of two vital components, i.e., learning and memory. Learning is the ability to get and process new information, whereas, memory refers to the storing ability of these information and to use it for future purposes. In several diseases, like AD and dementia, the cognition is altered based on severity or stage of the disease. The concept of cognition and the role of central cholinergic transmission in the acquisition of cognitive functions is well known since early sixties (Bartus et al., 1985; Holttum and Gershon, 1992). Several neurotransmitters are involved in the acquisition of learning and memory, but, the role of the cholinergic system is of greater curiosity for neurological researchers (Hagan and Morris, 1988). After the first ever evidence and report of cholinergic involvement in the cognition process by Deutsch, several new areas of neurological research including behavioral neuroscience, aging, dementia and behavioral pharmacology were emerged. Consequently, the deterioration of cholinergic neurons have been recognized to be involved in the cognitive deficits AD patients (Schliebs and Arendt, 2011). Furthermore, the antagonistic effect of non-selective anticholinergic agents like scopolamine has been recognized to aggravate memory deficit including attainment, retention, consolidation and recovery of memory (Stevens, 1981; Deiana et al., 2011). Thus, a boost in the cholinergic tone may potentially regress the cognitive hypofunction (Dumas and Newhouse, 2011; Haense et al., 2012; Anand et al., 2014). Based on this strategy, several drugs like ACh precursors, nicotinic and muscarinic agonists were tested. The idea failed due to limited efficacy, toxicity and bioavailability problems (Fisher, 2000; Mangialasche et al., 2010). Yet, AChE and BChE inhibitors were found effective in the management of AD.

Natural product based drugs including EOs and pure compounds are reported for effectiveness in numerous diseases (Ullah et al., 2015; Ayaz et al., 2016a,b, 2017). Several studies regarding the psychophysiological features of odors have been reported, though their cognitive functions are not fully implicit till now (Table 1). We also discussed several beneficial aspects of EOs in Alzheimer and dementia section using different memory models. In a study, Shimizu et al. (2008, 2009) reported the effect of EOs from *Eucalyptus globulus* Labill. and *Lavandula angustifolia* Mill. on sustained attention in a vigilance (condition of being attentive for a long period of time) task. In the lavender EO treated groups reaction time was significantly reduced in comparison to control group. Results of this study revealed that subjects treated with lavender EO sustained attention during prolong and exigent exercises. In a chronic study, the same group reported that sedative odors like lavender are more useful than stimulating odors in situations of tough exercises, whereas, too much alertness can harm vigilance. Moreover, stimulating odors were found more useful in coping less harsh tasks as they keep the subjects alert.

**Table 1:** Summary of Neuropharmacological studies conducted on essential oils and bioactive components.

Plant/Source	Part used	Study design	Results	References
*Polygonum hydropiper*	Leaves, Flowers EOs	AChE, BChE assays DPPH, ABTS, H_2_O_2_ assays	↓ AChE, BChE activity ↓ DPPH, ABTS, H_2_O_2_ radicals	Ayaz et al. (2015)
*Rumex hastatus* D. Don	Leaves EOs	AChE, BChE assays DPPH, ABTS, H_2_O_2_ assays	↓ AChE, BChE activity ↓ DPPH, ABTS radicals	Ahmad et al. (2016)
*Narcissus poeticus* L.	EOs	AChE, BChE inhibition	↓ AChE, BChE activity	Okello et al. (2008)
*Salvia leriifolia* Benth.	EOs	AChE, BChE inhibition DPPH assay Anti-inflammatory assay	↓ BChE activity ↓ DPPH, free radicals ↓ LPS-induced NO production	Loizzo et al. (2009)
*Marlierea racemosa* Vell.	EOs	AChE inhibition assay	↓ AChE activity	Souza et al. (2009)
*Cistus creticus*, *Cistus monspeliensis*, *Cistus salvifolius*, *C. villosus, C. libanotis*	EOs	AChE, BChE inhibition β-carotene bleaching FRAP assay	↓ AChE activity ↓ BChE activity ↓ Lipid peroxidation ↑ Reduced Fe3^+^-TPTZ	Loizzo et al. (2013)
*Eucalyptus globulus* Labill. *Lavandula angustifolia* Mill.	EOs	Sustained attention Tasks	↑ Vigilance ↓ Reaction time	Shimizu et al. (2008, 2009)
*Rosmarinus officinalis* L. *Mentha piperita*		Memory tasks Locomotor tasks	↑ Locomotor activity ↑ Motivate vigor ↑ Cortex stimulation ↑ Mood relaxation, Alertness	Hongratanaworakit (2009)
Orange, Coffee, Lavender, Liquorice	EOs/Fragrance	Visual attention	↑ Attention	Seo et al. (2010)
*Jasminum sambac* L	EOs	Topical application of EOS Aromatherapy effects on autonomic nervous system (ANS)	↑ Blood oxygen saturation ↑ Breathing rate ↑ Blood pressure ↑ Attention ↑ Behavioral arousal	Hongratanaworakit (2010)
Ginger EOs	6-gingerol	Aβ_25–35_ induced oxidative, nitrosative cells death in SH-SY5Y cells	↓ Aβ_25–35_ mediate cytotoxicity ↓ Apoptotic cell death ↓ DNA fragmentation ↓ Caspase-3 activity ↑ Bax/Bcl-2 ratio ↑ Iimmune glutathione	Lee et al. (2011)
SuHeXiang Wan	EOs	Administration of Aβ_1–42_ Oxidative stress in SH-SY5Y cells	↓ Aβ_1–42_ mediated JNK ↓ p38 ↓ Tau phosphorylation ↓ Apoptosis, ROS	Jeon et al. (2011)
Thymol, Carvacrol	EOS components	MWM test Aβ_25–35_ induce dysfunction	↓ Cognitive hypofunction ↑ Escape latency in MWM	Azizi et al. (2012)
*Coriandrum sativum* var. microcarpum		EPM, FST Reduced glutathione Antioxidant activity	↑ Locomotor activity ↓ Swimming, Iimmobility times ↑ Glutathione activity in HC	Cioanca et al. (2014)
*Zataria multiflora Boiss*.	EOs	Inj Aβ_25–35_ in CA1 region of HC, MWM task	↑ Escape Latency ↓ Quadrant entries	Majlessi et al. (2012)
*Lavandula angustifolia Lavandula hybrida* Rev.	EOs	Antioxidant, Anti-apoptotic study	↑ Superoxide dismutase (SOD) ↑ Glutathione per-oxidase ↑ Catalase (CAT) ↓ Reduced glutathione (GSH) ↓ lipid per-oxidation	Hancianu et al. (2013)
Thyme, Clove, Basil, Eucalyptus, Cinnamon leaf, Juniper, Chamomile	EOs, Thymol, Carvacrol	Antioxidant studies	↑ Free radicals scavenging effects	Tomaino et al. (2005), El-Ghorab et al. (2008) and Wei and Shibamoto (2010)
*Achillea millefolium*	EOs	*Ex vivo* anti-radicals	↓ lipid peroxidation	Candan et al. (2003)
*Lavandula angustifolia* Mill. and *Melissa officinalis* L.	EOs	Receptors binding study in dementia Sedative effects	↓ Radioligands binding to M_1_, 5HT_2A_, H_3_ and GABA_A_ receptors ↓ agitation	Elliott et al. (2007)
*Lavandula angustifolia* Mill.	EOs,	Electrophysiological tasks CCMAI, CNPI tasks	↓ TBPS binding to GABA_A_ ↔ AMPA, NMDA, AMPA receptors ↔ Cholinergic, nicotinic receptors ↓ CCMAI Score, CNPI ↓ Agitation behavior	Lin et al. (2007) and Huang et al. (2008)
*Lavandula angustifolia* Mill	Linalool	EPM Serum catecholamine Serum corticosterone	↔ GABA_A_ mediated anxiolysis ↑ Locomotor activity ↑ Motor functions	Cline et al. (2008)
Lemon EOs	Limonene, Perillyl alcohol	passive avoidance test Open field test	↑ Memory ↓ Dopamine ↓ AChE, BChE	Zhou et al. (2013)
*Cananga odorata*	EOs	Anxiety models	↓ Anxiety ↓ dopamine levels ↑ 5-HT	Gaydou et al. (1986) and Zhang et al. (2016)
Silexan capsules	Lavender EOs	HAM-A total score	↓ HAM-A sub-scores ↓ somatic, psychic anxiety	Woelk and Schläfke (2010)
*Citrus aurantium* L. *Citrus sinensis* L. Melaleuca alternifolia Nigella sativa L.	EOs	FST, Locomotor tasks Anxiety Animal models	↓ Anxiety ↑ Locomotor activity ↓ Escape latency	Chen et al. (2008), Murakami et al. (2009), Perveen et al. (2009) and Faturi et al. (2010)
*Crocus sativus* L.	Safranal	PTZ induced SE model	↓ GABA_A_ receptors ↓ Convulsions	Pathan et al. (2009)
*Ocimum basilicum* L.	EOs	Animal depression model PTZ and picrotoxin seizure tests	↓ Spontaneous activity ↓ Ataxia, Sedation, Ptosis ↑ Latency of convulsions episodes	Oliveira et al. (2009)
*Myristica fragrans* Houtt.		MES PTS seizures models	↓ Convulsions ↓ Onset of strychnine induced seizures ↔ Locomotor activity ↓ Partial, grand mal seizures ↔ Myoclonic, absence seizures	Wahab et al. (2009)

*↑: Increase/activate, ↓: Decrease/inhibit, ↔: No effect/not modulate. CCMAI, Cohen–Mansfield Agitation Inventory; CNPI, Chinese Neuropsychiatric Inventory; EOs, essential oils; 5-HT, 5-hydroxytryptamine; EPM, elevated plus maze; FST, forced swimming test; HC, Hippocampus; HAM-A total score, Hamilton Anxiety Rating Scale; PTZ, pentylene tetrazole; MWM, Morris water maze*.

*Rosmarinus officinalis* L. family Lamiaceae, is an ordinary household spicy plant and is frequently used in diet formulations owing to its strong anti-radical properties. The pharmacological actions of EO from *R. officinalis*, including, antibacterial, antifungal, anticancer, antioxidant and hypoglycemic are scientifically validated in several studies (Faixova and Faix, 2008). Moreover, rosemary EO are reported to stimulate the nervous system and thus perk up memory and concentration capacity. In a study, an improved performance was observed in a task due to the olfactory impact of rosemary EO with increase in overall quality of memory. The combination of EOs from *R. officinalis* and *M. piperita* were reported to augment the memory and activity level of mice and dogs. Rosemary EO also possess moderate AChE inhibitory activity and can synergistically act with 2-pinene and 1,8-cineole. It also increases locomotor activity, motivate vigor, stimulate cerebral cortex, cause mood relaxation and increase alertness (Hongratanaworakit, 2009). All these biological properties signify its potential use in the management of neurodegenerative diseases including AD, senile dementia myasthenia gravis.

It is well known that visual stimulus can influence olfactory perception, but less is known about the reverse case. In this regard, Seo et al. (2010) investigated the effect of fragrance on visual performance and attention. In the study design, four flavors including, orange, coffee, lavender and liquorice were presented to 60 healthy volunteers before and during a photographic slide show containing one harmonious and three dissimilar pictures. Eye tracking system was used to evaluate the level of visual attention in term of number and time of eye fixations after exposure to flavor. It is well known that the probands looked more frequent and longer at an object when they smell an odor, compared to situation without aroma. Results revealed that aroma promoted attention towards a consequent visual object when compared to non flavor situation. The nervous system stimulating effect of EO from jasmine (*Jasminum sambac* L) were tested by Hongratanaworakit (2010). Results revealed that jasmine aromatherapy increased autonomic mediated activities including blood oxygen saturation, breathing rate and blood pressure. An improved attention and subjective behavioral arousal was observed with more energetic and less sedative individuals of the aroma therapy group.

Owing to the memory enhancing capabilities of *Salvia lavandulifolia* Vahl (Spanish sage), Robbins and Broughan investigated the effect of EO from this plant on memory (Robbins and Broughan, 2007). Sixty volunteers were divided into three groups. The first group named “negative-expectancy group”, got special advice that *S. lavandulifolia* EO will impair their memory. The second group named “positive-expectancy group”, was delude to trust that *S. lavandulifolia* EO will have a positive influence on their memory. The third group, named “control group” was not informed about any benefits of test EO on their memory. Subsequent to the test, subjects were informed to participate in a second memory task which was analogous to the initial one. The main objective behind this study was to check the effect of expectations on actual outcome of therapy. As anticipated, the first group members were having less score in the second memory test as compared to the first memory test. Similarly, as per expectation, the second group (positive-expectancy) presented improved results in the second memory task in comparison to the first one. Amazingly, opposite to prophecy, members of the third group (control group) who did not have a verbal plan did not memorize an increased number of words in the second memory task. Results of the study re-confirmed that the effects of aromatherapy were in part based on psychological phenomena, especially expectancies, which could be seen particularly in matters of manipulation of expectations (Robbins and Broughan, 2007).

### Anti-Amyloid Efficiency of Essential Oils

**Figure 4:**
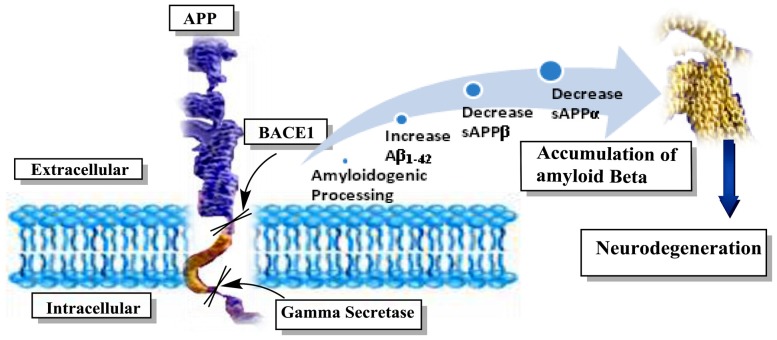
Schematic presentation of amyloidogenic pathway and formation of amyloid-β (Aβ) in AD. The amyloidogenic process is initiated by the enzymatic breakdown of amyloid precursor protein (APP) by beta amyloid cleaving enzyme (BACE1) called beta secretase at beta site. This is followed by catalytic cleavage of APP by gamma secretase to form non-soluble protein or Aβ. This Aβ accumulation in the neurons leading to impairment in the neurotransmission and neurodegeneration. EOs can inhibit the activity of BACE1 to hamper the Aβ load.

Among the pathological aspects of AD, the formation of cerebral plaques loaded with β-amyloid peptide (Aβ), congophilic (amyloid) angiopathy, dystrophic neuritis, appearance of NFTs in temporal lobe, loss of cholinergic neurons and white matter are important hallmarks (Haass and Selkoe, 2007). Aβ originates from enzymatic hydrolysis of an amyloid precursor protein called APP (Figure 4). The APP is cleaved by α, β and γ secretase enzymes in a sequence of steps, leading to the formation of Aβ_17–42_ catalyzed by α- and γ-secretases and the fabrication of neurotoxic Aβ_1–42_ via β and γ secretases (De Strooper et al., 2010). An imbalance among the generation and subsequent removal of Aβ results in the neuronal accumulation and can be a starting factor in the development of AD. Consequently, inhibition of beta amyloid cleaving enzyme (BACE1) is an imperative choice in management of AD owing to its role in the cleavage of the Aβ domain at the N-terminus in APP.

EOs and their active constituents were tested as potential anti-AD drugs. The anti-amyloid efficiency of these EOs were evaluated using cell lines and animal models. Aβ is implicated in the formation of senile plaques, an important pathological marker of AD, which cause apoptosis in neurons through oxidative or nitrosative stress. Lee et al. (2011) designed a study on the neuroprotective potentials and molecular mechanism of 6-gingerol, a predominant and pungent constituent of ginger EO against Aβ_25–35_ mediated oxidative and nitrosative cells death in SH-SY5Y cells. Pretreatment with 6-Gingerol provided protection against Aβ_25–35_ mediated cytotoxicity and apoptotic cell death. Cellular protection was mediated via inhibition of DNA fragmentation, interruption in mitochondrial membrane potential (MMP), activation of caspase-3 and increased Bax/Bcl-2 ratio. The neuroprotective mechanism of 6-gingerol was reported to be mediated via suppression of Aβ_25–35_ induced intracellular accretion of reactive oxygen species (ROS) and reactive nitrogen species (RNS). 6-Gingerol also reinstated the level of immune system antioxidant glutathione depleted by Aβ_25–35_. Moreover, the test sample up-regulated the expression of mRNA and proteins responsible for the synthesis of key enzymes like c-glutamylcysteine ligase, implicated in the biosynthesis of glutathione. The expression of above mentioned enzymes is mediated via activation of NF-E2-related factor. It is evident that 6-gingerol that 6-gingerol can be an effective remedy in the prevention and treatment of AD via enhancement of immune system antioxidant capacity. In another study, Jeon et al. (2011) investigated the EO from SuHeXiang Wan (SHXW) used as traditional medicine to treat seizures, infantile convulsions and stroke for beneficial effects in AD and other neurodegenerative disorders. Pre-inhalation of SHXW EO revitalized the memory of AD animal model injected with Aβ_1–42_. EO also suppressed Aβ_1–42_ mediated c-jun N-terminal kinase (JNK), protein kinases (p38) and tau phosphorylation in the hippocampus of animals. SHXW EO concealed Aβ-mediated apoptosis and ROS production by up-regulation of HO^-1^ and Nrf2 expression in SH-SY5Y cells. Results of this study suggested SHXW EO as a new inhalational therapy in the prevention and treatment of AD.

To scientifically validate the beneficial effects EO/components, Azizi et al. (2012) investigated the effects of thymol and carvacrol on cognitive function using animal models. In this study both test samples have improved cognitive functions in the animals injected with Aβ_25–35_ and scopolamine. The escape latency was increased in Morris Water Maze test (MWM) and the target quadrant entries were reduced. The Aβ_25–35_ and scopolamine induced memory impairment were reversed following administration of thymol and carvacrol. During toxicity studies both samples were found safe even at extremely higher concentrations. Results of this study provided important evidence regarding the effectiveness and safety of thymol and carvacrol in amyloid induced disorders like AD and dementia. Anti-amyloid, cholinesterase inhibitory, anti-inflammatory and antioxidant potentials of these EO components may be responsible for beneficial effects in cognitive hypo-function.

Cioanca et al. (2014) tested EO from coriander, *Coriandrum sativum* var. microcarpum for its potential antidepressant, anxiolytic and antioxidant potentials in inhalation form using Aβ_1–42_ rat models of AD. The anxiolytic and antidepressant-like effects of inhaled coriander volatile oil were studied by means of elevated plus-maze (EPM) and forced swimming test (FSM) models. Also, the antioxidant activity in the hippocampus was assessed using catalase (CAT) specific activity and the total content of the reduced glutathione (GSH). EO treatment significantly improved locomotor activity, reduced swimming and immobility times within FSM animals pre-treated with Aβ_1–42._ Furthermore, EO inhalational therapy augmented the level of glutathione in the hippocampus of the animal models and the activity of CAT enzymes signifying its antioxidant potentials. The current study suggests that multiple inhalational exposures to coriander EO markedly improve anxiety, depression and relieve oxidative stress in AD. In another study, Majlessi et al. (2012) investigated the EO of *Zataria multiflora* Boiss. (Lamiaceae) for cognitive and neuroprotective effects in animal models of AD. Results of the study revealed that a concentration dependent improvement in the cognitive abilities of the animals as indicated by various parameters like increase in escape latency, traveled distance, heading angle were observed in Aβ_25–35_ pre-treated groups. The EO was highly safe in acute toxicity test even at high concentrations than the tested dose. The beneficial effects of EO were attributed to the presence of components effective in inflammation, oxidative stress and inhibitors of cholinesterase’s.

### Role in Coping Oxidative Stress

**Figure 5:**
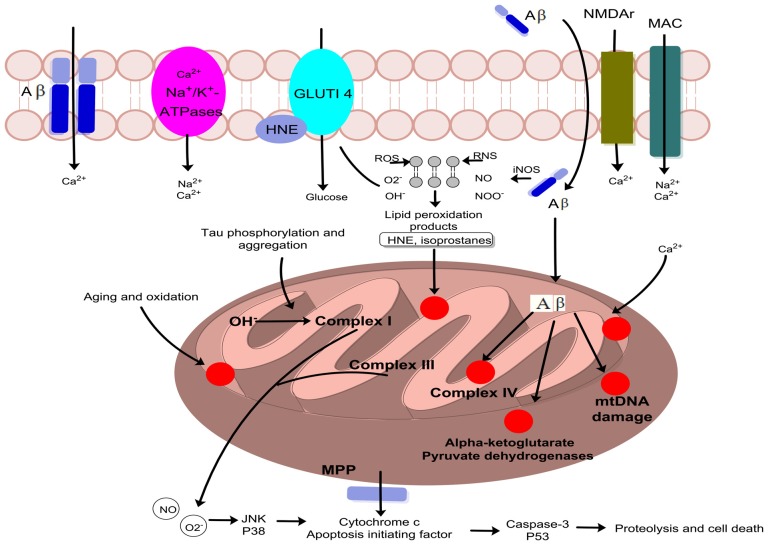
The figure summarize various sources of free radicals production with special focus on Aβ as initiator of reactive oxygen species (ROS) and reactive nitrogen species (RNS). After generation, the free radicals attack membrane lipids, cellular organelles which leads to the production of mitochondrial toxins hydroxynonenal (HNE) and malondialdehyde. Oxidative stress damage membrane-bound ion-selective ATPases and stimulate calcium influx via stimulation of NMDA receptors, membrane attack complex (MAC), and ion-specific Aβ pore formation with ultimate increase in cytosolic and mitochondrial calcium load. Cellular amyloid targets cytochrome c oxidase, α-ketoglutarate and pyruvate dehydrogenase and thus cause mitochondrial DNA damage causing its fragmentation. Lipid peroxidation products enhance phosphorylation and aggregation of tau proteins which subsequently inhibit complex I. Excessive quantities of ROS and RNS are produced at complexes I and III. Further, the mitochondrial membrane potential (MMP) crumple and permeability-transition pores (ψm) opened leading to activation of caspases. Aβ also stimulate the production of stress-induced protein kinases (p38) and c-jun N-terminal kinase (JNK), in addition to p53, which stimulate apoptosis leading to cellular damage.

**Figure 6:**
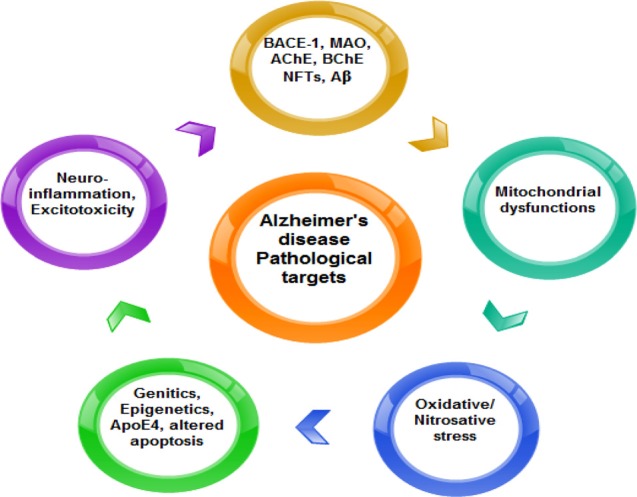
Summary of the pathological targets in AD.

Free radicals are generated during aerobic respiration, an essential aspect of living beings. These free radicals readily attack fatty acids, DNA, proteins, other essential molecules and are implicated in a variety of disorders including AD, cancer, aging, inflammation, Parkinson’s disease, diabetes, atherosclerosis and liver disease (Ayaz et al., 2014; Ahmad et al., 2015; Kamal et al., 2015; Sadiq et al., 2015; Shah et al., 2015). Free radicals which are produced during oxidation process are neutralized to non-radical forms by enzymes including CAT and hydroperoxidase. However, when free radicals generation is abnormally high or the immune system is depleted, then administration of free radicals scavengers from outside is necessary (Halliwell, 1994; Ullah et al., 2016). Furthermore, in AD patients and aging brain, mitochondrial dysfuntion lead to excessive production of oxidizing free radicals which lead to oxidative stress with subsequent oxidative damage and pathological abnormalities. Beta Amyloid (Aβ) is a potent instigator of ROS and RNS. These reactive species rapidly initiate oxidative damage of neural, microglial, cerebrovascular cells and tissues (Engel et al., 2008, as shown in Figures 5, 6).

Lavender is an important traditional medicine traditionally used in Asia, Europe, Rome, ancient Greece and can also be found in ancient Jewish texts and Bible. It is used as alternative remedy for the treatment of inflammation, headache, stress and depression. In order to investigate the neuroprotective effects of lavender, Hancianu et al. (2013) conducted a study describing the antioxidant and antiapoptotic potentials of EO from lavender (*Lavandula angustifolia* ssp. *Angustifolia* Mill. and *Lavandula hybrida* Rev). Lavender EO displayed significant antioxidant and antiapoptotic potentials in scopolamine-induced dementia rat models. Sub-acute inhalational exposure to EO significantly augmented the level of immune system antioxidant enzymes including superoxide dismutase (SOD), glutathione per-oxidase and CAT. Furthermore, total amount of GSH and lipid per-oxidation were reduced in specific brain tissues homogenates. Thus protective actions of lavender EO can be mediated via strong antioxidant activities. In addition, DNA cleavage prototypes were not present in EO treated groups, signifying anti-apoptotic potentials of the samples. Overall, results of the study indicate that strong antioxidant and anti-apoptotic activities of EO are major contributing factors in the neuroprotective action of the test samples.

Recent advances in neurological research revealed that BACE1, also known as secretase, catalyze the cleavage of APP leading to the formation of ß-amyloid peptides. Being amyloidogenic in nature, these Aβ accumulate in the AD brain and provoke inflammatory responses with subsequent liberation of free radicals causing neuronal damage (Nitsch, 1996; Asai et al., 2007; Vassar, 2014). Antioxidant agents may be useful in AD chemotherapy by attenuating the inflammatory pathways through scavenging of free radicals (Kamal et al., 2015). Recently, plant based therapies have got considerable attention owing to their comparative safety, potency and efficacy on multiple targets. Among the natural products, EOs from aromatic and medicinal plants are of great interest owing to their antioxidant, cholinesterase inhibitory, anti-amyloid properties and high availability at the target due to lipophilic character. Thus, EOs can be very effective and alternative for the management of AD in comparison to synthetic drugs which are associated with severe side effects (Brahmachari, 2013).

Numerous EOs have been reported to possess strong antioxidant potentials and can be effectively used in free radicals induced disorders including neurological diseases and aging. For instance, EOs from thyme, clove, eucalyptus, cinnamon leaf, juniper, basil, chamomile, coriander, cumin are reported to possess considerable antioxidant potentials (Tomaino et al., 2005; El-Ghorab et al., 2008; Wei and Shibamoto, 2010). *Thymus spathulifolius* is reported with strong anti-radicals activity owing to the presence of active constituents thymol and carvacrol at high concentrations of 36.5% and 29.8%, respectively (Tepe et al., 2004). Likewise, the antioxidant capability of Egyptian corn silk was attributed to the presence of high concentrations of carvacrol (58.1%) and thymol (20.5%) (El-Ghorab et al., 2008). Dietary intake of oregano oil has been reported to significantly delay lipid oxidation in animal models (Botsoglou et al., 2004). EO from *Achillea millefolium* were reported to scavenge hydroxyl free radicals via inhibition of lipid peroxidation in the liver tissue homogenate of animals (Candan et al., 2003). In another study, EOs from *Salvia multicaulis* and *Salvia cryptantha* demonstrated antioxidant activities higher than the standard drugs (Tepe et al., 2004).

Many aroma components of the terpenoids and terpenes groups like α-terpinene, β-terpinolene, 1,8-cineole, β-terpinene, menthon, thymol, isomenthone, linalool and eugenol which are abundant in EOs are reported to responsible for antioxidant potentials (El-massry and El-Ghorab, 2006; Wei and Shibamoto, 2010). Likewise, EOs from *Thymus mastichina*, *Thymus caespititius* and *Thymus camphorates* were observed to be highly effective against free radicals owing to the presence of bioactive components linalool and 1,8-cineole. Similarly, the EO of *Melissa officinalis* L. which is enriched in menthone, isomenthone, geranial and citronellal is reported as highly effective antioxidant remedy (Mimica-Dukic et al., 2004). These studies support the idea that EOs are highly potent antioxidants, which are biodegradable, lipophilic, comparatively safe and can be potential substitutes for synthetic drugs in the management of neurodegenerative disorders (Yanishlieva-Maslarova and Heinonen, 2001).

### Role in Dementia

Several EOs were tested for the treatment of agitation and other symptoms of dementia in search for more useful drugs as neuroleptic agents, are associated with unwanted side effects and limited efficacy. For instance, Elliott et al. (2007) employed EOs from *Lavandula angustifolia* Mill. and *Melissa officinalis* L. belonging to Lamiaceae for the management of agitation in individuals with severe dementia. The sedative and calming effect of both EOs is already established which can contribute in consolidation of memory. In the receptor binding capability study, both oils extensively inhibited radioligands binding to the muscarinic M_1_, 5HT_2A_, histamine H_3_ receptors and GABA_A_ receptor channel site. *M. officinalis* EO displayed broad receptor binding capacity in comparison to *L. angustifolia* EO, and showed affinity for binding with 5HT_1A_ and the agonist binding site of GABA_A_ receptors. The results of this study revealed that both EOs act as substrate for and interact with several receptors, and can be effectively used to relieve the symptoms of agitation. Conversely, *M. officinalis* EO has got the ability to reduce social withdrawal times and increased the time of constructive activities of dementia patients. In a study, Huang et al. (2008) investigated the electrophysiological properties of EOs with focus on modulation of ion channels. Lavender EO inhibited binding of *t*-butyl bicyclophosphorothionate (TBPS) to GABA_A_ receptors channel of the rat forebrain. Yet no effect on AMPA, NMDA, AMPA, cholinergic and nicotinic receptors was observed. *L.angustifolia* and *M. officinalis* EOs in combination (50:50) has inhibited flunitrazepam binding. In electrophysiological task, *L. angustifolia* EO reversibly inhibited GABA mediated current in rat cortical cultures, revealed that lavender oil reversibly with no inhibitory effect on AMPA and NMDA induced currents. Furthermore, *M. officinalis* EO inhibited the binding of TBPS to GABA_A_ receptor channel, however no effect on AMPA, NMDA, cholinergic and nicotinic receptors was observed. Electrophysiological tests showed that balm EOs could reversibly inhibit GABA mediated currents. However, no inhibitory effects on AMPA and NMDA induced currents were observed (Abuhamdah et al., 2008).

The effect of EO from *L. angustifolia* on agitation behaviors in dementia patients was reported by Lin et al. (2007). Elder individuals with AD, vascular dementia or other types of dementia were included in the study. Seventy patients were divided in two groups, the aroma group maintained on lavender EO for 3 weeks followed by administration of odorless sunflower inhalation for 3 weeks, whereas the second group did the diametrically opposite. The clinical response was evaluated in terms of Chinese version of the Cohen–Mansfield Agitation Inventory (CCMAI) and Neuropsychiatric Inventory (CNPI). Results of the study revealed that the average CCMAI and the mean CNPI scores were significantly reduced after the aromatherapy with lavender. Moreover, the lavender therapy was well tolerated during the course of therapy and a significant decline in agitation behavior was observed. Consequently, lavender aromatherapy could be a useful alternative to psychotropic drugs (Lin et al., 2007). Limonene from the EO of lemon were tested by Zhou et al. (2009) in scopolamine induced dementia model applying passive avoidance test and open field test. Limonene and its metabolite perillyl alcohol exhibited significant improvement in memory. The level of neurotransmitters especially dopamine was lower in scopolamine treated animals, but this effect was overturned by limonene or perillyl alcohol therapy prior to the scopolamine injection. These components of lemon EO also displayed *in* *vitro* cholinesterase inhibition activity (Zhou et al., 2013).

## Other Neuropharmacological Actions of Essential Oils

### Anxiolytic Potentials of Essential Oils

Anxiety is a state of psychological and physiological disturbances manifested by cognitive, emotional, behavioral and somatic elements. All together, these factors provoke an unpleasant sensation coupled with apprehension, fret, disquiet and restlessness. The onset of anxiety is sudden and unexpected without any triggering stimulus and thus is a serious medical state. In order to cope the unusual panic situation, the body is liable to some symptoms, like tension, sweating, palpitations, chest pain, papillary dilatation and shortness of breath (O’Connor et al., 2000; Borkovec and Ruscio, 2001). The current anxiolytic agents like benzodiazepines are associated with numerous side effects, like drug tolerance, abuse and sedation. Consequently, aromatherapy with psychoactive EOs may be a useful alternative therapy to relieve anxiety (Woelk and Schläfke, 2010). Several plants EOs have been reported to possess strong anxiolytic potentials. For instance, *L. angustifolia* Mill (lavender), *Citrus sinensis* L. (orange), *Santalum album* RBr (sandal wood), *Rosa damascena* Mill (rose), *Citrus bergamia* Risso. (bergamot), *Salvia sclarea* L. (clary sage), *Anthemis nobilis* L (roman chamomile) and Pelargonium species EOs are strong anxiolytic agents (Setzer, 2009; López et al., 2017). The chemical composition and effects of EOs, constituents from *Cananga odorata* (ylang-ylang) were evaluated by researchers in animal models using behavioral assessment tools (Gaydou et al., 1986; Zhang et al., 2016). Furthermore, the level of neurotransmitters and their metabolites were also assessed subsequent to oil exposure. *C. odorata* EO exhibited considerable anxiolytic effect in animal models. The constituents of *C. odorata including* benzyl benzoate, linalool and benzyl alcohol also displayed anxiolytic effect in animal model of anxiety. EOs constituents lowered the dopamine levels in striatum and augmented the level of 5-hydroxytryptamine (5-HT) in hippocampus of experimental animals.

Among the anxiolytic phytotherapies, the lavender EO and its active component linalool is most frequently used frequently explored. The aniolytic action of linalool was investigated by Cline et al. (2008) using EPM and analysis of serum catecholamine and corticosterone levels. Results of the study revealed that linalool lake GABA_A_ mediated anxiolysis, but locomotor activity and motor functions were improved in animal models. In a randomized double blind study, the anxiolytic effects of orally administered lavender EO were tested on 97 human volunteers using neutral and anxiety provoking film clips. Effects of EO on mood, anxiety, positive and negative effects scale, heart rate, state trait anxiety inventory and galvanic skin responses were measured following ingestion of 100, 200 μl lavender EO capsules. Results showed that 200 μl lavender EO reduced the symptoms of anxiety in individuals exposed to neutral films. However, in anxiety-provoking film groups, lavender EO exerted mild beneficial effects. This study concludes that lavender EO is effective in mild to moderate anxiety but is not effective in severe anxiety (Bradley et al., 2009).

Pharmacologically effective aromatherapy can be a better choice to relieve anxiety in patients petrified of surgical interventions or dental procedures. In a study, relative efficacy of lavender EO and standard drugs were tested in pre-operative anxiety patients (Braden et al., 2009). Volunteers (150 individuals) were divided into control group kept on standard drugs, test/aroma group maintained on EOs (lavender) and non-aroma group treated with standard drug plus jojoba oil. The anxiety symptoms were evaluated on admission and transfer to operating room at pre-and post therapy stages. Lavender EO treated groups displayed significant decline in the anxiety symptoms after transfer to operating rooms, thus signifying its potential role in relieving pre-operative anxiety. Another preparation of lavender EO (Silexan capsules) were tested by Woelk and Schläfke (2010) in a controlled clinical trial using benzodiazepine as standard drug. Patients were kept on either test drug or lorazepam for 6 weeks. The level of anxiety was objectively assessed by the Hamilton Anxiety Rating Scale (HAM-A total score) from baseline till last therapy. Results revealed that EO based therapy was able to relieve the symptoms of generalized anxiety and was analogous to the standard drug lorazepam in efficacy. In EO and larazepam treated groups, somatic, psychic anxiety and two HAM-A sub-scores were significantly reduced. Furthermore, sub-scores including Self-rating Anxiety Scale, Clinical Global Impressions of Severity Disorder, the Penn State Worry Questionnaire and a sleep diary exhibited similar positive efficiency in both therapies. Lavender EO based therapy was devoid of sedation, potential drug abuse and well tolerated by participants. Thus, it can be a better, safe-yet effective alternative to benzodiazepines therapy in the management of generalized anxiety (Woelk and Schläfke, 2010).

Shirodhara an Ayurvedic oil therapy medicated with sesame oil, is commonly known remedy for anxiety and effective in the management of altered state of consciousness (ASC). To evaluate the pharmaco-physio-psychological effects of Shirodhara, lavender EO were added to the formulation. Volunteers were divided into three groups namely, plain Shirodhara group (sesame oil), aroma-Shirodhara (0.3% lavender EO + sesame oil) and control group. The oils were applied via a robotic oil-dripping system. Different parameters including heart rate, anxiety, temperature of hand and feet and ASC were recorded. Strong anxiolytic and ASC effects were observed in aroma group. The significance between anxiolysis-ASC and psychological effects increased foot skin temperature were more obvious in the aroma Shirodhara group as compared to other groups. Authors concluded that the psycho-physiological effects of lavender-Shirodhara were based on relaxing effects of lavender EO via olfactory nerves, better absorption of oils via skin and physiological effects of sesame oil dripping on the forehead mediated by the somatoautonomic reflex through thermosensors or pressure sensors using the trigeminal cranial nerve (Xu et al., 2008).

Neroli oil is another traditional anxiolytic remedy obtained from the flowers of *Citrus aurantium* L (bitter orange). To scientifically validate its anxiolytic effects, Chen et al. (2008) investigated the neroli EO via inhalation to the animal models. The level of anxiety was assessed using FSTs and locomotors tasks both in aroma and standard control groups. Both EOs based therapy and standard drug (alprazolam) exhibited anxiolytic activities in behavioral tests, though the exact anxiolytic mechanism was not explained. This study provided a scientific base for the potential use of neroli oils in the management of anxiety disorders. Other EOs, including *Citrus sinensis* L. (sweet orange aroma), *Melaleuca alternifolia* Maiden and Betche, Alpinia zerumbet (shell flower) and *Nigella sativa* L. (black seeds) were investigated in detail using animal models. These EOs exhibited significant anxiolytic activities (Murakami et al., 2009; Perveen et al., 2009; Faturi et al., 2010; Satou et al., 2010).

The constituents of EOs are also of great interest to the scientific community dealing with neurological disorders. For instance, the effect of inhaled linalool on anxiety was investigated by Linck et al. (2009). Beneficial effects of linalool were assessed in terms of influence on social interactions, anxiety, aggressive behavior and memory. Social interactions were significantly improved and aggressive behavior were greatly reduced following treatment with linalool. Great anxiolytic effect was observed in treated animal models as indicated by spending more time in light area during light/dark test. However, an unwanted effect on memory was observed at high a dose which was attributed to anxiolytic and relaxant actions of linalool. Likewise, the beneficial effects of monoterpene phenol carvacrol on behavioral disturbances were analyzed using animal models. Results confirmed strong anxiolytic action of carvacol without effecting the locomotor activity of the test animals (Melo et al., 2010).

The effect of aromatherapy on cancer-induced anxiety is reported by several researchers. According to literature, 13.9%–25% cancer patients suffer from anxiety (about 70% non-pathological) disorders which greatly affect their life style and chemotherapy (Mantovan et al., 2009). *Citrus bergamia* Risso EO are commonly used as aromatherapy to treat symptoms of cancer pain, stress-induced anxiety and mood disturbances. While analyzing the neuropharmacological effects of EO from *C. bergamia*, an Italian research group reported that these EO liberated exocytic and carried mediated amino acids release beside neurotransmitters in mammalian hippocampus. These results supported the assumption that these EO interfere with normal and pathological synaptic plasticity. These results and some neuroprotective effects of EO from *C. bergamia* signify its use in alternative medicine for anxiety disorders (Imanishi et al., 2009; Bagetta et al., 2010). Several other studies also reported the significance of aromatherapy in cancer-induced anxiety disorders (Hansen and Hansen, 2007; Chang, 2008; Imanishi et al., 2009).

### Role in Epilepsy and Convulsions

Epilepsy is a neuronal disorder characterized by chronic and persistent neuronal activity as a result of reduced seizures threshold in the CNS (Thews et al., 1999). Among the suggested mechanism is the excessive release of an excitatory neurotransmitter glutamate which after binding with glutamatergic neurons causes the excessive release of calcium at the postsynaptic neuronal cells. Another theory explains the disorder in terms of mutations that lead to the production of ineffective inhibitory peptide called GABA. It is effecting 50 million populations worldwide and the disease cannot be eradicated completely. Epilepsy has more than 40 types, classified on the bases of age of onset, type of seizures, type of therapy and prognosis. About 30% patients are presented with uncontrolled seizures despite of using clinically available anti-epileptic drugs (Mischel et al., 1995; Cendes, 2005). Status epileptics (SE) is the most dangerous type of epilepsy characterized by persistent and recurrent episodes of seizures for more than 30 min and is associated with greater mortality rates.

Several studies were reported on the therapeutic effectiveness of EOs as an alternative to the currently available drugs for the management of epilepsy. The antianticonvulsant activity of safranal, a monoterpene aldehyde and active aromatic constituent of *Crocus sativus* L. was investigated by Pathan et al. (2009). Results revealed that safranal exhibited a dose dependent anticonvulsant activity in pentylene tetrazole (PTZ) mediated SE models. The proposed anticonvulsant mechanism of this volatile component was attributed to its agonistic activity on GABA_A_ receptors. Thus, safranal was proposed as a potential future complementary therapy in the management of SE. The monoterpenoid alcohol component of several plant’s EOs, terpinen-4-ol, is reported for effectiveness in convulsions (de Almeida et al., 2011). Animals treated with terpinen-4-ol exhibited significant decline in tonic hind paws convulsions and spontaneous motor activity, whereas, the waiting period of seizures was increased.

The EO of a common household spice *Ocimum basilicum* L. and related species were investigated by Oliveira et al., owing to the already reported anticonvulsant and CNS depressant actions (Oliveira et al., 2009). Chemical analysis revealed the presence of psychoactive components, 1,8-cineole, geraniol and linalool. The EO exhibited CNS depressant effects via decline in spontaneous activity, ataxia, sedation and ptosis at all tested doses. Furthermore, the EO showed a significant prolongation in sleeping period and reduced the sleep latency. These EOs increased the latency of convulsions episodes in both PTZ and picrotoxin seizure tests. Results of this study concluded that *O. basilicum* EOs possess anticonvulsant and CNS depressant potentials mediated via central GABAergic receptors. Wahab et al. (2009) reported the anticonvulsant efficiency of EO from *Myristica fragrans* Houtt. (nutmeg) using various animal models of seizures. Generally, the onset of anticonvulsant effect was quick but duration of action was brief, still it exhibited a significant anticonvulsant effect in MES model of the disease. In PTZ induced convulsions models, a dose dependent anticonvulsant effect was observed even after the jerks were started. Nutmeg EO delayed the onset of strychnine mediated seizures did not altered locomotor activity even at high concentrations. In conclusion, nutmeg EO might be an effective remedy in the treatment of partial and grand mal seizures. However, it is not effective in myoclonic and absence seizures owing to its slight potentiating effects of clonic seizures. In a neuropharmacological study of *Cymbopogon proximus* EO, El Tahir and Abdel-Kader (2008) observed that partial-to-complete protection was offered in PTZ, strychnine, picrotoxin and electric shock induced convulsions models. *Pimpinella anisum* L. (anise) is traditionally effective in epilepsy, though the mechanism of its anti-epileptic activity is not clearly understood. In a study, Janahmadi et al. (2008) evaluated the anti-epileptic effect of EO in pre and post PTZ-induced seizures models. Results revealed that anise EO cause neuronal hyper-excitability by reducing after-hyperpolarization in snails. Thus, anise EO must be carefully used in individuals suffering from epilepsy (Janahmadi et al., 2008).

## Conclusions

In the current review article, we have only focused on the research work related to our topic published in peer reviewed journals. Researchers from various regions studied EOs in an attempt to rationalize their traditional uses or to discover alternative therapies to the current drugs and to reduce side effects associated with the use of current medications. *In vitro*, *in vivo* and clinical studies were performed on EOs and volatile constituents to find their efficacy and mechanism of action. Concluding the current literature based review, it is noted that EOs are effective on almost all currently known pathological targets of AD. EOs also possess neuroprotective, anti-aging potentials and are effective in dementia, epilepsy, anxiety and other neurological disorders. Regarding AD, it is important that EOs which are effective on multiple targets (multi-potent agents) must be screed to find more effective drugs in comparison to the currently available drugs which have limited efficacy and are useful for symptomatic relief only. Anti-aging EOs will be more effective in the prevention of these neurological disorders. Special focus must be on the edible EOs which are either part of diet or used as spices will be more useful. Special concern regarding the kinetic profile, route of administration and dose are important tasks in the development of EOs as new drugs.

## Author Contributions

MA conceived the idea, carried out literature survey and drafted the manuscript. AS helped in chemistry of essential oils and corrected the final version of the manuscript. MJ, FU, FS, JA provided useful guidelines, technical support at every step of the project to which this data belong and edited the manuscript. All authors read and approved the final manuscript for publication.

## Conflict of Interest Statement

The authors declare that the research was conducted in the absence of any commercial or financial relationships that could be construed as a potential conflict of interest.

## Abbreviations

Aβ: amyloid beta
ABTS: 2,2-azinobis 3-ethylbenzthiazoline-6-sulfonic acid
ACh: acetylcholine
AChE: acetylcholinesterase
AD: Alzheimer disease
APP: amyloid precursor protein
ASC: altered state of consciousness
BACE1: beta amyloid cleaving enzyme
BChE: butyrylcholinesterase
CAT: catalase
CCMAI: Cohen–Mansfield Agitation Inventory
CNPI: Chinese Neuropsychiatric Inventory
CNS: central nervous system
DPPH: 1,1-diphenyl 2-picrylhydrazyl
EOs: essential oils
GC-MS: gas chromatography-mass spectrometry
GSH: reduced glutathione
JNK: c-jun N-terminal kinase
MMP: mitochondrial membrane potential
NFTs: neurofibrillary tangles
NMDA: N-methyl-d-aspartate
p38: protein kinases
ψm: permeability-transition pores
PgP: pglycoproteins
PTZ: pentylene tetrazole
RNS: reactive nitrogen species
ROS: reactive oxygen species
SE: status epileptics
SHXW: SuHeXiang Wan
SOD: superoxide dismutase
TBPS: t-butyl bicyclophosphorothionate.
